# Online asynchronous decoding of error-related potentials during the continuous control of a robot

**DOI:** 10.1038/s41598-019-54109-x

**Published:** 2019-11-26

**Authors:** Catarina Lopes-Dias, Andreea I. Sburlea, Gernot R. Müller-Putz

**Affiliations:** Institute of Neural Engineering, Graz University of Technology, Graz, Austria

**Keywords:** Biomedical engineering, Cognitive control

## Abstract

Error-related potentials (ErrPs) are the neural signature of error processing. Therefore, the detection of ErrPs is an intuitive approach to improve the performance of brain-computer interfaces (BCIs). The incorporation of ErrPs in discrete BCIs is well established but the study of asynchronous detection of ErrPs is still in its early stages. Here we show the feasibility of asynchronously decoding ErrPs in an online scenario. For that, we measured EEG in 15 participants while they controlled a robotic arm towards a target using their right hand. In 30% of the trials, the control of the robotic arm was halted at an unexpected moment (error onset) in order to trigger error-related potentials. When an ErrP was detected after the error onset, participants regained the control of the robot and could finish the trial. Regarding the asynchronous classification in the online scenario, we obtained an average true positive rate (TPR) of 70% and an average true negative rate (TNR) of 86.8%. These results indicate that the online asynchronous decoding of ErrPs was, on average, reliable, showing the feasibility of the asynchronous decoding of ErrPs in an online scenario.

## Introduction

Brain-computer interfaces (BCIs) are systems that measure brain activity, often using electroencephalography (EEG), and convert it into actions of an external device^1^. As BCIs enable communication without movement, they are a valuable tool to provide more independence to people with severe motor disabilities^2–4^.

The main obstacle to the widespread use of BCIs is their non-optimal performance, which sometimes leads to a misinterpretation of the user’s intention and a consequent execution of a wrong action. The user’s experience with the BCI can be spoiled by occurrence of many mistakes or by the effort to correct them.

The user’s awareness of the committed mistake is associated with a neural pattern named error-related potential (ErrP). ErrPs occur both in humans and in monkeys and can be measured using several imaging techniques^5–12^. Additionally, ErrPs morphology is comparable in humans with and without spinal cord injury^13^. ErrPs are related with conflict monitoring^14^ and have been reported in association with the awareness of self-committed mistakes, observed mistakes of another person or agent, and BCI’s mistakes^14–17^.

The use of ErrPs is an intuitive approach to improve BCIs’ performance, either in a corrective manner, by allowing the BCI to take a corrective action, or in an adaptive manner, by reducing the possibility of future errors^18–20^.

The detection of ErrPs in a time-locked manner is well established^21–23^, and it has been extensively applied in discrete BCIs, whose actions occur in a discrete manner, allowing users to interact with a computer or with a robot^24–31^.

Recently, an effort has been made to develop BCIs that provide a more intuitive control to the user, by e.g., providing continuous control to the user^32–34^. In this situation, the user can perceive, at any moment, that an error occurred. This possibility triggered the research on the asynchronous detection of ErrPs^35–39^.

In the current study, we investigate the feasibility of the online asynchronous ErrPs’ detection, while participants continuously controlled a robotic arm towards a target, using their right hand. In 30% of the trials, the user’s control of the robot was halted at random point. Participants could regain the robot’s control if an ErrP was detected after the error onset. To our knowledge, this is the first report of online asynchronous detection of ErrPs.

## Materials and Methods

### Participants

15 right-handed volunteers (5 women) participated in the experiment. All participants had normal or corrected-to-normal vision and had no history of brain disorders. The participants were, on average, 23.5 ± 2.5 years old (mean ± std). Participants were paid 7.50 euros per hour, were explained the experimental protocol and signed an informed consent form that had been previously approved by the local ethics committee of the Medical University of Graz (Ethical approval number 30-275 17/18). The experiment was performed in accordance with the Declaration of Helsinki.

### Hardware and measuring layout

We recorded EEG and EOG data at a samplig rate of 500 Hz using BrainAmp amplifiers and an ActiCap cap (Brain Products, Munich, Germany). We used 61 EEG electrodes and 3 EOG electrodes. The EOG electrodes were placed above the nasion and below the outer canthi of the eyes. The ground electrode was placed at position AFz and the reference electrode was placed on the right mastoid. The layout of the EEG electrodes is described in Fig. 1 of the Supplementary Material.

### Experiment layout

**Figure 1:**
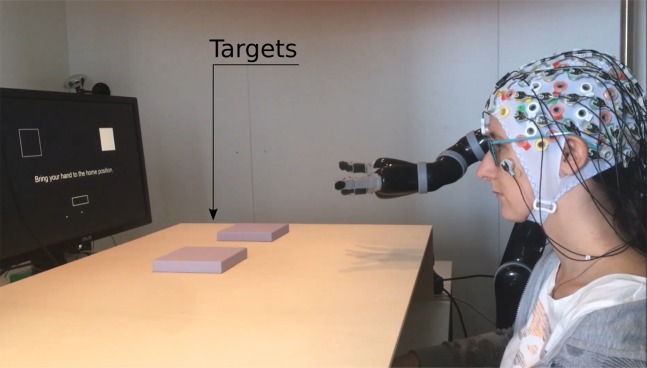
Experimental setup. In this figure, the robot is at its home position. The squares on the screen represent the physical targets (violet cuboids) on the wooden structure. The rectangle on the bottom part of the screen represents the participant’s hand home position and the text above it states ‘Bring your hand to the home position’. Inside the wooden structure, there is a Leap Motion device (not visible here) used to track the participants’ right hand movement.

Figure 1 depicts the physical layout of the experiment. Participants sat on a chair in front of a table. On the table was a wooden structure: 4-sided box, with open sides towards the participants and the tabletop. On the ceiling of the structure was a Leap Motion device (Leap Motion, San Francisco, United States), used to track the participants’ right hand (not visible in Fig. 1). The participants kept their right hand lying on the table, inside the wooden structure. This setup occluded the participants’ hand from their field of view. On the right side of the participants, we placed a robotic arm (Jaco Assistive robotic arm - Kinova Robotics, Bonn, Germany). On the wooden structure, were placed two physical targets: violet cuboids with a square base of 14 cm side. The centres of the targets were 35 cm apart and their mid-point was located 30 cm in front of the home position of the robot’s hand, as shown in Fig. 1. Behind the structure, within the participant’s line of sight to the targets, was a monitor that displayed information regarding the experiment. The participant shown in Fig. 1 gave her informed consent for the photo to be made available in an open-access publication.

### Controlling the robot

During the *trials*, participants could control the position of the robot’s hand on a horizontal plane, by moving their hand on the table, which was tracked with the Leap Motion. To reduce the range of the participants’ movements, we considered the robot’s hand displacement to be three times larger than the participants’ hand displacement.

### Experiment overview

Before the experiment, two blocks in which participants performed eye movements were recorded. The experiment then consisted of 12 blocks of 30 trials each. 70% of the trials of each block were *correct trials* (21 trials) and the remaining 30% were *error trials* (9 trials).

The sequence of correct and error trials within each block was randomly generated using a uniform distribution to place the error trials. We defined a maximum of 2 consecutive error trials in each block and repeated the randomization procedure until the sequence of trials satisfied this condition.

Half of the trials in each block were associated with the left target and the remaining trials with the right target. The sequence of targets within each block was randomly assigned using a uniform distribution. We defined a maximum of 3 consecutive trials with the same target in each block and repeated the randomization procedure until the targets’ sequence satisfied this requirement.

### Pre-trial

**Figure 2:**
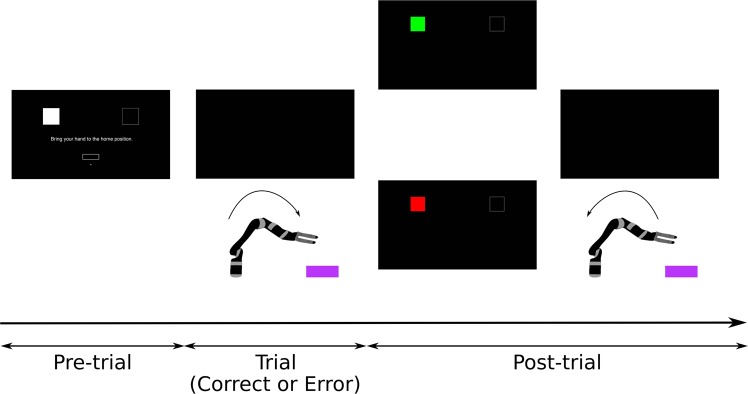
Experimental protocol. During the pre-trial period, participants could rest for as long as they wished. The pre-trial period ended and a new trial started when the participants moved their hand to its home position. During the trials, the screen was black. Participants were instructed to bring the robot’s hand to the selected target. A trial finished either when the robot reached the target or after 6 seconds, in case target was not reached. Afterwards (post-trial period), the squares reappeared on the screen for 1.2 seconds and gave feedback regarding hitting the target: a green square indicated that the target was hit and a red square indicated that the target was not hit. Then, the screen turned black, the robot automatically returned to its home position and a new pre-trial period started.

During the pre-trial period, the monitor displayed information regarding the coming trial. As depicted in Figs. 1 and 2, on the top part of the screen were displayed two squares representing the targets lying on the wooden structure. One of the squares was filled in white and the other had no fill. The filled square indicated the selected target for the coming trial. On the bottom part of the screen was a rectangle, representing the home position of the participant’s hand. The position of the participant’s hand was depicted by a dot on the screen. The trial would start when the dot entered the rectangle. This ensured that the participant’s hand was at a comparable position at the beginning of each trial (within a 1 × 3 cm rectangle).

Participants could use the pre-trial period to rest for as long as they needed. When participants felt ready to start the trial, they had to bring their hand below the home position, fixate their gaze on the physical target and finally enter the rectangle from below. This final step ensured a forward movement of the robot. Participants were also instructed to keep their gaze fixed at the target during the entire trial duration, in order to prevent eye movements.

### Trials

The aim of each trial was to bring the robot’s hand from its home position to the selected target. During the trials, the screen was black. A trial ended when the robot’s hand was above the intended target (hit) or after 6 seconds (no hit). Afterwards (post-trial period), as shown in Fig. 2, the two squares from the pre-trial period reappeared on the screen for 1.2 seconds and the filled square was now coloured in either green (hit) or red (no hit). This feedback was always in line with the behaviour of the robot. Then, the screen would turn black, the robot would automatically return to its home position and a new pre-trial period would start.

#### Error Trials

During these trials, the paradigm triggered an *error*. The error consisted in interrupting the participants’ control of the robot and adding a 5 cm upwards displacement to the robot’s hand. Participants perceived the error by noticing the robot stopping and lifting. The errors occurred randomly, when the robot’s hand was within 6 to 15 cm in the forward direction from its home position. This represents approximately 25 to 65% of the minimal forward displacement necessary for the robot to hit the target. For every error trial, we drew a value *d*_*e*_ from a uniform distribution U($[6,15]$). The error was triggered when the robot’s hand reached the distance *d*_*e*_ cm, in the forward direction, from its home position.

#### Correct Trials

In these trials, the paradigm did not trigger any error. Participants reached the selected target in 99.75 ± 0.14% of the correct trials (mean ± std). Correct trials lasted on average 2.02 ± 0.14 s (mean ± std). Correct trials were comparable in the *calibration and online parts of the experiment*.

### Calibration and online parts of the experiment

The *calibration part* of the experiment comprised the first 8 blocks and the *online part* comprised the last 4 blocks. The calibration part was used to collect data to *train an ErrP classifier* and to find a *threshold* for the classifier. In the online part of the experiment, we tested the ErrP classifier, tuned with the calculated threshold, for the asynchronous detection of ErrPs.

For a matter of fluidity of the experiment, we decided not to give participants feedback of the false positive detections, i.e., of the *ErrP detections* when no error had occurred. Thus, from the participants’ perspective, the online ErrP classifier had no effect on the correct trials and affected only the error trials. However, false positive detections were taken into account when evaluating the classifier.

#### Calibration error trials

In the error trials during calibration, when the error happened, the participants lost control of the robot, which remained still for the rest of the trial. The total trial duration was 6 seconds. Participants were instructed not to move until the trial ended.

#### Online error trials

In the error trials during the online part of the experiment, the participants had the possibility of correcting the robot’s errors. If, after the error onset, an ErrP was detected by the ErrP classifier (true positive detection), the robot’s hand lowered 5 cm and the participants regained control of the robot. The downward movement informed the the participants of the ErrP detection. Participants were instructed to move the robot’s hand to the selected target when regaining control of the robot. To accommodate the extra movement, we added 6 seconds to the maximal trial duration when the first true positive detection occurred. When no true positive detection occurred, the robot remained still, as in the error trials during calibration.

#### Correct trials

For the participants, correct trials were identical in both the calibration and the online parts of the experiment, due to our decision of not giving feedback of the false positive detections in the online part of the experiment.

### Data preprocessing

Eye movements and blinks were removed from the EEG data, using the data recorded right before the beginning of the experiment and using the subspace subtraction algorithm^40^. The EEG signal was then filtered between $[1,10]\,{\rm{Hz}}$ using a causal Butterworth filter of order 4.

#### Defining events

For the calibration error trials, we defined the error onset as the moment in which the robot started its upwards displacement. The error onset was individually calculated for every error trial, based on the robot’s position. The average delay between the error marker and the error onset was 0.210 ± 0.004 s (mean ± std).

For the online error trials, we considered an average error onset, by adding the average delay of the robot, calculated from the calibration data (0.210 s), to the time of the error marker in every error trial. This aimed to compensate the less reliable error onset estimation in case an ErrP occurred between the error marker and the start of the robot’s upwards displacement (false positive detection).

Correct trials were not associated with any intrinsic event. Therefore, we defined a virtual onset, occurring one second after the start of every correct trial. The virtual onset was chosen at a time-point in which errors could already occur in the error trials, in order to assure a comparable expectation in the participants.

### Electrophysiological analysis

For the electrophysiological analysis, we considered an EEG epoch of 1.5 s from every trial. For the correct trials, we considered the interval $[\,-\,0.5,1.0]\,{\rm{s}}$, time-locked to the virtual onset (0 s). For the calibration error trials, we considered the interval $[\,-\,0.5,1.0]\,{\rm{s}}$, time-locked to the error onset (0 s). For the online error trials, we considered the interval $[\,-\,0.5,1.0]\,{\rm{s}}$, time-locked to the average error onset (0 s).

### Detection of error-related potentials

We used the data from the calibration part of the experiment to build an ErrP classifier that was tested asynchronously in the online part of the experiment.

#### Train an ErrP classifier

For every participant, we considered all trials from the calibration part of the experiment. We took, as features, the amplitudes of all 61 EEG channels at every time-point within a 450 ms window of every trial. The window started 300 ms after the error onset of error trials and 300 ms after the virtual onset of correct trials.

Next, in order to reduce the number of features, we performed principal component analysis (PCA) on the features, keeping the components that explained 99% of the data’ variability. These components were then used as features to train a shrinkage-LDA classifier with two classes: error and correct. After PCA we kept, on average, 139.5 ± 13.5 features per participant (mean ± std). Figure 2 of the Supplementary Material depicts the grand-average original feature space in the time-spatial domain as well as the grand-average projection into the time-spatial domain of the features kept after PCA.

#### ErrP detection

The classifier was constructed to be evaluated in an asynchronous manner, using a sliding window, with a leap of 18 ms. The classifier’s evaluation of each window resulted in the probability of the analysed window to belong to either class (correct or error). We defined an *ErrP detection* when two consecutive windows had a probability of belonging to the error class above a certain threshold *τ*.

#### Threshold *τ* for the ErrP classifier

The threshold *τ* was obtained using the calibration data and used in the online part of the experiment to tune the ErrP classifier.

In order to find the threshold that best suited each participant, we performed a 2 × 5-fold asynchronous cross-validation in the participant’s calibration data, where we tested 41 thresholds: from 0 to 1 in steps of 0.025. We used a low number of repetitions in the cross-validation to promote a shorter duration of the experiment.

As evaluation metric for the asynchronous ErrP detection in the cross-validated data, we defined the true negative trials (TN trials) as the correct trials in which no ErrP was detected during the entire trial duration. We defined the true positive trials (TP trials) as the error trials in which no ErrP was detected before the error onset and at least one ErrP was detected within 1.5 s of the error onset.

Then, we calculated the average true negative rate (TNR) and the average true positive rate (TPR) for all the tested thresholds, based on the 10 iterations. The average TNR and average TPR were further smoothed using a moving average with 7 samples. The smoothed curves were named moving average TPR and moving average TNR.

For every participant, we considered the threshold that maximized performance to be the one that maximized the product of the moving average TPR and the moving average TNR. This threshold was then used in the online part of the experiment.

### Online ErrP detection

The ErrP classifier was tested online in the last 4 blocks of the experiment. We decided to relax the evaluation metrics when testing the classifier online (in comparison with the metrics described for the cross-validated data) in order to consider the possible occurrence of secondary error-related potentials^28^.

In the online evaluation, we defined the true negative trials (TN trials) as the correct trials in which no ErrP was detected (keeping the same definition used in the evaluation of the cross-validated data). Additionally we now defined the true positive trials (TP trials) as the error trials in which no ErrP was detected before the average error onset and at least one ErrP was detected after the average error onset.

A video of the online experiment can be seen in the Supplementary Material. The participant in the video gave her informed consent for it to be made available in an open-access publication.

## Results

### Electrophysiology

#### Calibration

**Figure 3:**
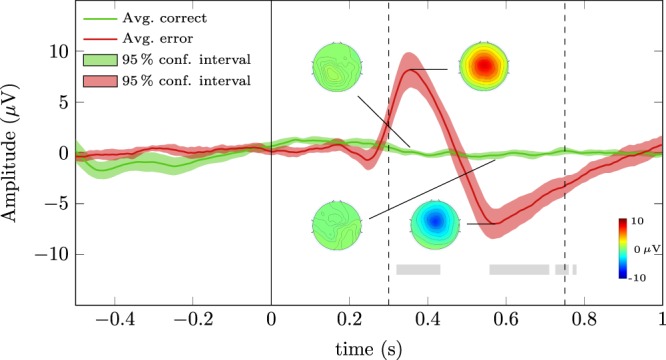
Grand average correct and error signals of the calibration part of the experiment at channel FCz (green and red solid lines, respectively). The green and red shaded areas represent the 95% confidence intervals of the grand average signals. The regions in which correct and error signals were significantly different are marked with a grey rectangle (Wilcoxon rank-sum tests, Bonferroni corrected, $p < 0.01$). The vertical line at *t* = 0 s represents the error onset of error trials and the virtual onset of correct trials. The dashed vertical lines at *t* = 0.30 s and *t* = 0.75 s delimit the window used to train the ErrP classifier.

Figure 3 depicts the grand average correct and error signals of the calibration part of the experiment at channel FCz (green and red solid lines, respectively). The green and red shaded areas represent the 95% confidence intervals of the grand average signals. The time-intervals in which correct and error signals were significantly different ($t=[0.320,0.432]\,{\rm{s}}$, $t=[0.558,0.710]\,{\rm{s}}$, $t=[0.726,0.760]\,{\rm{s}}$ and $t=[0.770,0.780]\,{\rm{s}}$) are represented by grey rectangles (Wilcoxon rank-sum tests, Bonferroni corrected, $p < 0.01$). The vertical line at *t* = 0 s represents the error onset for the error trials and the virtual onset for the correct trials. The error signal presents a small negativity with peak amplitude −0.71 μV at 0.246 s, followed by a positivity with peak amplitude of 8.46 μV at 0.354 s, which is followed by a broader negativity with peak amplitude −6.98 μV at 0.568 s. Figure 3 also depicts the topoplots of correct and error trials at the time-points *t* = 0.354 s and *t* = 0.568 s.

#### Online part

**Figure 4:**
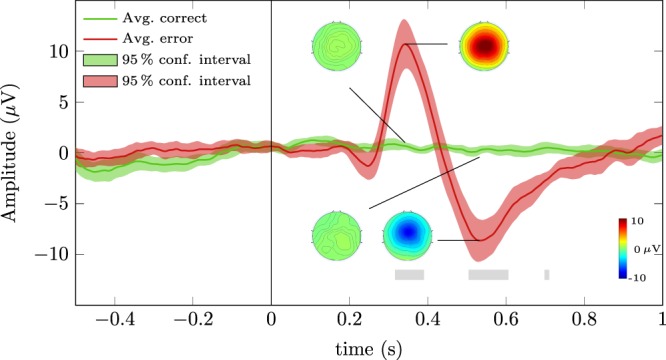
Grand average correct and error signals of the online part of the experiment at channel FCz (green and red solid lines, respectively). The green and red shaded areas represent the 95% confidence interval for the grand average signals. The grey rectangles represent the time-intervals in which correct and error signals were significantly different (Wilcoxon rank-sum tests, Bonferroni corrected, $p < 0.01$). The vertical line represents the average error onset of the error trials and the virtual onset of the correct trials.

Figure 4 depicts the grand average correct and error signals of the online part of the experiment at channel FCz (green and red solid lines, respectively). The green and red shaded areas represent the 95% confidence intervals of the grand average signals. The time-intervals in which correct and error signals were significantly different ($t=[0.316,0.390]\,{\rm{s}}$, $t=[0.504,0.606]\,{\rm{s}}$ and $t=[0.698,0.710]\,{\rm{s}}$) are represented by grey shaded areas (Wilcoxon rank-sum tests, Bonferroni corrected, $p < 0.01$). The error signal presents a small negativity with peak amplitude −1.29 μV at 0.246 s, followed by a positivity with peak amplitude 10.7 μV at 0.342 s and by a broader negativity with peak amplitude −8.63 μV at 0.532 s. Figure 4 also depicts the topoplots of correct and error trials at the time-points *t* = 0.342 s and *t* = 0.532 s.

### Asynchronous ErrP detection

#### Offline asynchronous ErrP detection in the calibration data

During the experiment, we performed asynchronous ErrP detection in the calibration data to find the threshold *τ* that was used online (using a 2 × 5-fold cross-validation to reduce the experiment duration, as described in section *Threshold τ for the ErrP classifier*).

**Figure 5:**
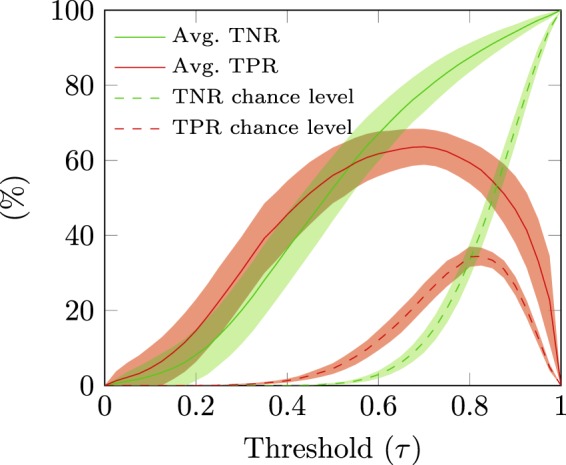
Asynchronous ErrP detection in the calibration data. Grand average TNR and TPR (solid green and red lines, respectively) in function of the threshold *τ*, calculated from the 10 × 5-fold cross-validation in the calibration data. The chance-level TPR and TNR are represented with red and green dashed lines. The shaded areas represent the 95% confidence intervals for the grand average curves.

For visualization purposes, here we present the asynchronous ErrP detection results, obtained using a 10 × 5-fold cross-validation in the calibration data, in which we tested 41 thresholds *τ* from 0 to 1, with steps of 0.025. The evaluation metric used to assess the results was the same as described in section *Threshold τ for the ErrP classifier*. Figure 5 displays the grand average TPR and TNR of the asynchronous classification performed using a 10 × 5-fold cross-validation in the calibration data (red and green solid lines, respectively), in function of the threshold *τ*. The chance-level TPR and TNR (red and green dashed lines, respectively) were obtained by performing the same classification procedure with randomly permuted training labels. The shaded green and red areas represent the 95% confidence intervals of the grand average curves. The obtained TPR results were significantly higher than chance levels TPR results for thresholds $\tau \in [0.100,0.975]$ (Wilcoxon rank-sum tests, one sided, Bonferroni corrected, $p < 0.01$). The obtained TNR results were significantly higher than chance level TNR results for thresholds $\tau \in [0.150,0.975]$ (Wilcoxon rank-sum tests, one sided, Bonferroni corrected, $p < 0.01$).

#### Online asynchronous ErrP detection

**Figure 6:**
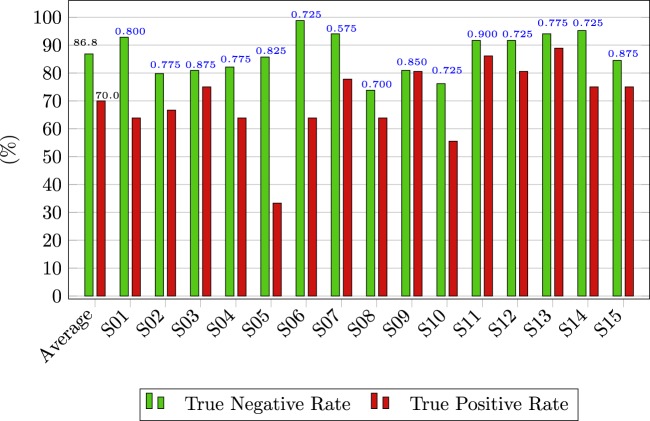
Online asynchronous ErrP detection. The green bars represent the TNR of every participant and their average. The red bars represent the TPR of every participant and their average. The average TPR was 70.0% and the average TNR was 86.8%. The blue numbers indicate the threshold *τ*, used for each participant.

In the online part of the experiment, we used for the asynchronous ErrP detection, a subject specific-threshold *τ*, calculated as described in section *Threshold τ for the ErrP classifier*. The evaluation metric used to assess the results was described in section *Online ErrP detection*. Figure 6 depicts the TPR and TNR of the online asynchronous ErrP classification for every participant as well as the average results. We obtained an average TPR of 70.0% and average TNR of 86.8%. The blue numbers on top of the bars indicate the used threshold *τ* used for every participant.

**Figure 7:**
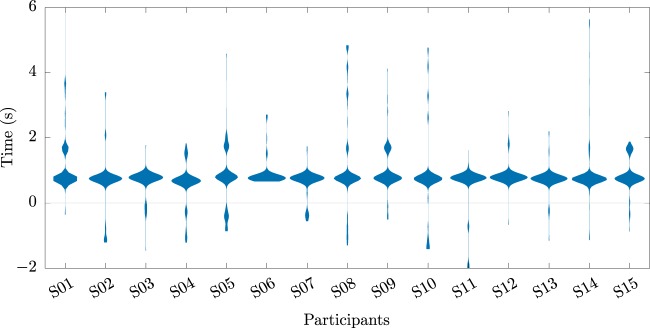
Time-points of all ErrP detections in the online scenario. Violin plots, for every participant, of the time-points of all ErrPs detections in the error trials of the online part of the experiment, in relation to the average error onset (*t* = 0 s).

Figure 7 depicts, for every participant, a violin plot of the time-points of all the ErrP detections in the error trials of the online part of the experiment, in relation to the average error onset (*t* = 0 s).

## Discussion

In the described experiment, we asynchronously decoded ErrPs in an online scenario. Here, we showed the ErrPs’ electrophysiology during the calibration and the online parts of the experiment. In both conditions, ErrP displayed similar shapes but the grand average ErrP in the online condition exhibited stronger peak amplitudes.

We chose to display, in both conditions, the ErrPs’ electrophysiology using EEG signals filtered with a causal filter in order to match the ErrPs’ appearance in the online scenario. The displayed results differ from standard state-of-the-art literature, in which it is commonly used a zero-phase filter. In our situation, the typical N200 component of ErrPs is shifted to after the ErrPs’ P300 component. The difference is a direct consequence of using a causal filter and does not reflect any particularity of the neural activity.

We also showed results regarding the asynchronous ErrP detection in the calibration data using cross-validation, where different thresholds for the ErrP classifier, ranging from 0 to 1, could be tested.

Finally, we displayed the results of the asynchronous ErrP detection for the online part of the experiment, in which we obtained an average TNR of 86.8% of and an average TPR of 70%. In the online part of the experiment, all participants displayed a major cluster of ErrP detections within one second of the error onset, as shown in Fig. 7. Some participants displayed a secondary cluster of ErrP detections, which can possibly be associated with secondary ErrPs, as described by Salazar-Gomez and colleagues^28^. Alternatively, these later detections could also be possibly linked to an event-related potential associated with the robot resuming its movement (that the classifier erroneously classified as an ErrP).

We decided not to give participants feedback regarding false positive detections, neither in correct nor in error trials, to maintain the flow of the experiment and avoid interruptions. Still, from Figs. 6 and 7, we can infer that the majority of ErrP detections were not associated with false positive detections.

Literature supports that, in general, feedback improves BCIs performance and several feedback modalities have been tested^41–45^. But, to the best of our knowledge, the effect of ErrPs’ feedback has not been studied yet. Nevertheless, we believe it can help participants to be more engaged and could possibly be associated with the increase in the peak amplitudes of the ErrP verified in the online scenario. Moreover, we believe that providing feedback of the false positive detections could help participants to understand if they have any control over these detections and, if so, adapt their behaviour accordingly.

Therefore, we conclude that the asynchronous decoding of ErrPs in an online scenario is possible and reliable and we suggest that giving participants full feedback of the ErrP detections would not decrease and would possibly increase participants’ performance.

## Supplementary information

Video of the experiment

Supplementary material

## Data Availability

The datasets generated during and/or analysed during the current study are available from the corresponding author on reasonable request.
