# Supplementary material for: Online asynchronous decoding of error-related potentials during the continuous control of a robot
Source: Sci Rep. 2019 Nov 26;9:17596. doi: 10.1038/s41598-019-54109-x (PMC6879530; doi:10.1038/s41598-019-54109-x)
Supplement: Supplementary file 2 — Supplementary material [file 41598_2019_54109_MOESM2_ESM.pdf]

ONLINE ASYNCHRONOUS DECODING OF ERROR-RELATED POTENTIALS DURING THE CONTINUOUS CONTROL OF A ROBOT

CATARINA LOPES-DIAS, ANDREEA I SBURLEA AND GERNOT R MÜLLER-PUTZ

Figure 1 shows the location of the 61 EEG electrodes used in the experiment.

Figure 2 shows that, in the classification procedure, the principal components retained after PCA preserve the activity of the original feature space.

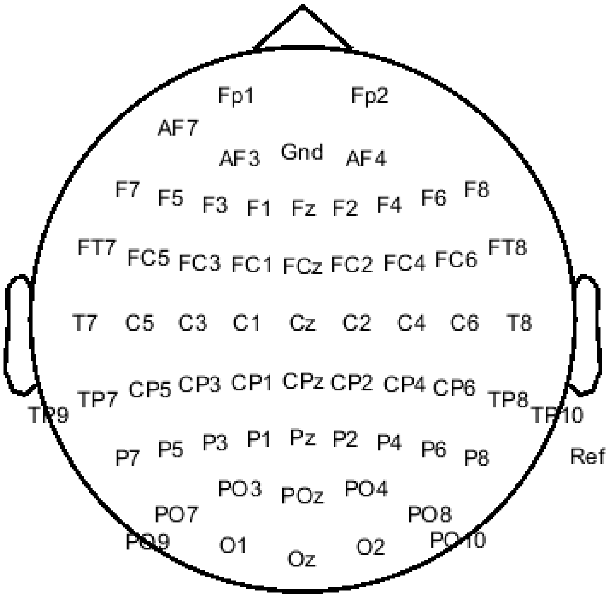

FIGURE 1. Layout of the EEG electrodes. The ground electrode was placed at position AFz and the reference electrode was placed on the right mastoid.

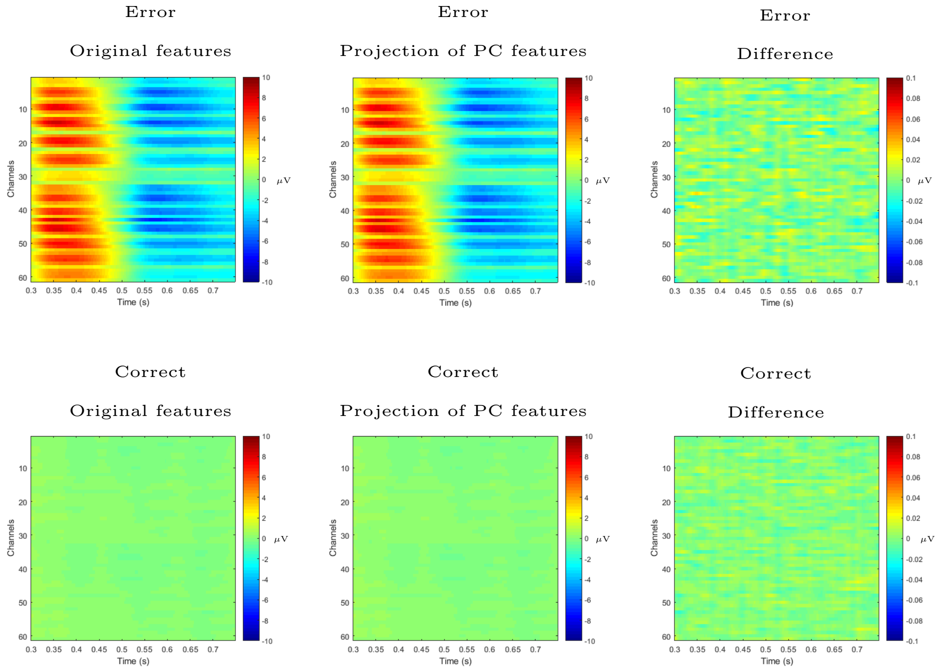

Figure 2. Grand average classifier features: Left: Grand-average original feature space. Middle: Grand-average projection into the temporal-spatial domain of the principal component (PC) features retained after PCA. Right: Difference between the grand-average original feature space and the grand-average projection of the features retained after PCA. The channel order is the following: 1-Fp1, 2-Fp2, 3-F7, 4-F3, 5-Fz, 6-F4, 7-F8, 8-FC5, 9-FC1, 10-FC2, 11-FC6, 12-T7, 13-C3, 14-Cz, 15-C4, 16-T8, 17-TP9, 18-CP5, 19-CP1, 20-CP2, 21-CP6, 22-TP10, 23-P7, 24-P3, 25-Pz, 26-P4, 27-P8, 28-P09, 29-O1, 30-Oz, 31-O2, 32-PO10, 33-AF3 34-AF4 35-F5, 36-F1, 37-F2, 38-F6, 39-FT7, 40-FC3, 41-FC4, 42-FT8, 43-FCz, 44-C5, 45-C1, 46-C2, 47-C6, 48-TP7, 49-CP3, 50-CPz, 51-CP4, 52-TP8, 53-P5, 54-P1, 55-P2, 56-P6, 57-PO7, 58-PO3, 59-POz, 60-PO4, 61-PO8.
